# Native Mass Spectrometry in Fragment-Based Drug Discovery

**DOI:** 10.3390/molecules21080984

**Published:** 2016-07-28

**Authors:** Liliana Pedro, Ronald J. Quinn

**Affiliations:** Eskitis Institute for Drug Discovery, Griffith University, Brisbane 4111, Queensland, Australia; liliana.pedro@griffithuni.edu.au

**Keywords:** native MS, fragment-based drug discovery, noncovalent interaction, protein-ligand complex, fragment-based screening, binding stoichiometry, binding specificity, binding affinity, structure-activity relationship

## Abstract

The advent of native mass spectrometry (MS) in 1990 led to the development of new mass spectrometry instrumentation and methodologies for the analysis of noncovalent protein–ligand complexes. Native MS has matured to become a fast, simple, highly sensitive and automatable technique with well-established utility for fragment-based drug discovery (FBDD). Native MS has the capability to directly detect weak ligand binding to proteins, to determine stoichiometry, relative or absolute binding affinities and specificities. Native MS can be used to delineate ligand-binding sites, to elucidate mechanisms of cooperativity and to study the thermodynamics of binding. This review highlights key attributes of native MS for FBDD campaigns.

## 1. Introduction

In 1989 the scientific community witnessed what was thought to be impossible: “flying elephants”, as was later described by John Fenn in his Nobel Lecture [1]. The introduction of the soft ionization method, electrospray ionization (ESI), by John Fenn and his colleagues, made possible the transfer of a protein (or other macromolecule) in a solution into the gas-phase as ions with no molecular fragmentation [2]. This achievement, along with the invention of matrix-assisted laser desorption/ionization (MALDI) [3,4], opened up a whole new world of applications in the field of mass spectrometry (MS), resulting in enormous progress and mass spectrometry becoming an outstanding analytical method.

**Figure 1:**
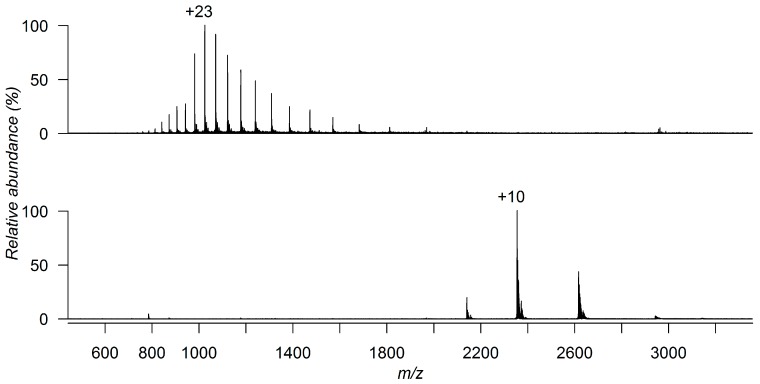
ESI-MS spectrum of *Plasmodium vivax* guanylate kinase (*Pv*GK) under denaturing conditions (50:50 (*v*/*v*) acetonitrile-water, 1% formic acid) (**top**) and native conditions (10 mM ammonium acetate solution) (**bottom**).

Soon after native MS was born, in 1990, Brian T. Chait and co-workers demonstrated that the charge state distributions (CSD) of proteins produced by ESI were related to the folding state of the proteins in the solution [5]. For instance, they showed that electrosprayed cytochrome c molecules assumed wider distributions of charge states centered around higher charge states when sprayed from pH 2.6 than from pH 5.2 aqueous solutions, arguing this resulted from differences in the conformational states of the protein in solution. Indeed, today, it is known that the solution conditions that are needed to maintain a folded protein are not the same as the optimal conditions for standard ESI operation. For maximal sensitivity, a solution with pH of 2–4 for positive ion ESI is usually used for protein analysis. This is accomplished by the addition of traces of an organic acid (e.g., formic acid) and an organic modifier, such as methanol or acetonitrile. Under these conditions, the protonated protein generates a mass spectrum that has a wide distribution of multiply charged ions. However, these solution conditions are not generally tolerated when trying to maintain a protein in its folded state, as proteins are normally denatured in solution at a pH value outside the pH 6–8 range [6,7]. To avoid denaturation, the sample has to be formulated in or dialyzed against a buffer that is compatible with ESI-MS and allows maintenance of the protein’s native conformation. Ammonium salts (ammonium acetate and ammonium hydrogen carbonate) offer fairly high volatilities and allow the study of proteins under physiological conditions (pH 6–8). The advantage of using a volatile buffer instead of water alone is that the buffer ensures that electrochemical effects in the nebulizer cone do not affect solution pH [8]. Under these conditions, the protein can remain in its native, or folded state, and the mass spectrum generated exhibits a narrower distribution of charge states with the protein carrying fewer charges [9] (Figure 1).

Ganem et al. were the first to demonstrate that molecules that were noncovalently associated in solution could be transferred into the gas-phase with ESI and detected as an intact complex [8,10]. In 1991 (just two years after the development of ESI-MS), a published paper reported the receptor-ligand interaction between FK-binding protein and FK506 [11]. That same year, Katta and Chait reported ESI-MS detection of the intact globin-heme interaction of myoglobin [12] and Ganem et al. reported the enzyme-substrate pairing of lysozyme and a hexasaccharide [13]. Soon after these initial reports, the technique was exploited for the determination of dissociation constants (K_D_) [14]. The first example of the application of the direct ESI-MS assay to quantify protein-ligand affinities was reported in 1993 by Loo and co-workers [15]. Another important contribution was by Smith and co-workers, who characterized noncovalent complexes of various modified benzensulphonamide inhibitors and carbonic anhydrase (CA) by ESI-FT-ICR (Fourier Transform-Ion Cyclotron Resonance) [16]. Their work demonstrated, for the first time, that the affinity of multiple potential ligands for a single macromolecular target could be evaluated simultaneously using mass spectrometry, and laid the foundation for subsequent screening approaches [10].

Many of the early studies of noncovalent complexes used triple-quadrupole mass spectrometers [8,17]. Even though the maximum acquisition range of these instruments has been improved, the low resolving power at high *m*/*z* represented a considerable disadvantage [8,9]. In contrast, time-of-flight (TOF) mass analyzers have a theoretically unlimited mass range and can achieve high mass resolution and sensitivity. Hybrid instruments of the quadrupole-time-of-flight geometry were then developed, exploiting the *m*/*z*-range benefits of time-of-flight with the selection abilities of a quadrupole [8]. Mass spectrometers with other types of analyzer have also been developed and used for the analysis of protein complexes. Fourier transform ion cyclotron resonance (FT-ICR) is a versatile instrument that has great mass accuracy and resolution. Another boost for the analysis of macromolecular protein complexes was the introduction of nano-ESI. By comparison with standard ESI, which uses flow rates of a minimum of 2 µL/min to achieve a consistent spray of ions, nano-ESI only requires flow rates in the range of sub-µL/min. Consequently, it consumes less sample and increases sensitivity, by the generation of smaller droplets, which increase the ionization and desolvation efficiencies [7,9].

Today, native MS can be used not only to detect ligand binding to specific targets, but also to determine binding affinity, stoichiometry and specificity [7,18,19]. Native MS can be exploited as a method for ligand binding site identification [20,21,22,23,24,25], for elucidation of mechanisms of cooperativity [26,27,28] and for studying the thermodynamics of ligand binding [29,30,31]. This review highlights key attributes of native ESI-MS that are valuable for any fragment-based drug discovery (FBDD) campaign. Concerns commonly raised about this technique and challenges inherent to its application in FBDD are discussed.

## 2. Preserving Native or Near-Native Protein Conformations in the Gas-Phase

This is probably the most important premise of native MS, and yet the most controversial. The native conformation of a protein is its fully folded and biologically functional form. While, in native MS, the observation that a protein gives a lower and narrower CSD than when sprayed from denaturing solutions is an indication of a folded conformation, it does not necessarily mean that the native solution-phase conformation has been maintained in the gas-phase [32,33,34]. In solution, the noncovalent interactions responsible for a protein secondary, tertiary and quaternary structure are governed by the distance between the interacting species, the dielectric constant (ε) of the medium and, in the case of ionic species, their charges. In the settings of MS, the consequences of going from an aqueous environment (ε = 80) to vacuum (ε = 1), comprise an increase of Coulombic/electrostatic interaction energies by a factor of 80 and of the vdW interactions by a factor of 6400 [7]. Hydrophobic interactions, which in solution are driven by the entropy gain when structurally organized solvent molecules are liberated from a hydrophobic interface, are lost [32]. Moreover, solvent removal eliminates the competition of water for hydrogen bonds within protein tertiary structures [35]. Possible differences between the gas and solution-phase pH can also alter the ionization state of determined species [36]. Therefore, it is not unlikely that, depending on the nature of the protein and level of energetic activation during the ESI process and ion transfer from atmospheric pressure to vacuum, the noncovalent interactions present in solution undergo rearrangement, leading to a different gas-phase conformation.

**Figure 2:**
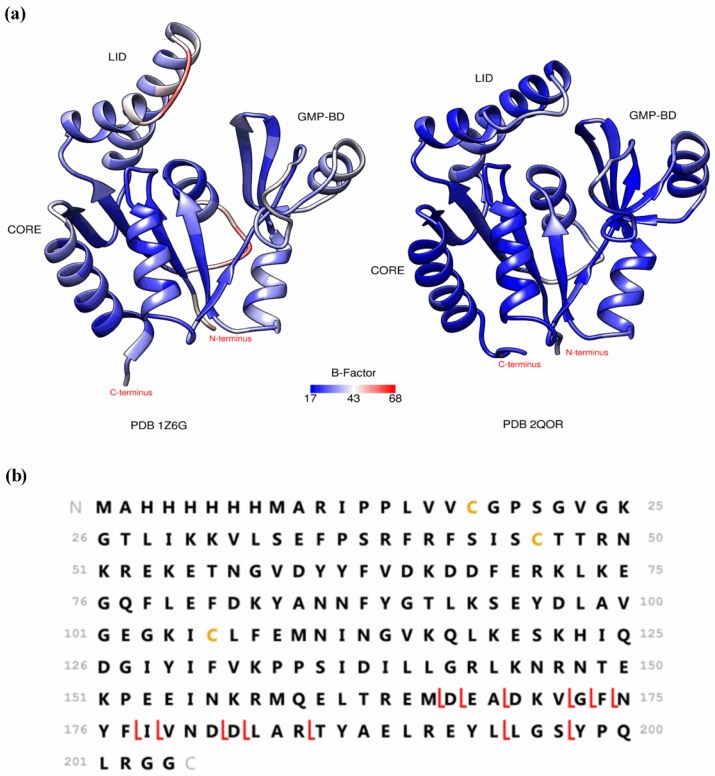
(**a**) Crystal structure of *Plasmodium falciparum* guanylate kinase (*Pf*GK) (PDB 1Z6G) and *Pv*GK colored by average residue B-factor; (**b**) amino acid sequence of *Pv*GK and the assigned cleavage sites after ECD of *Pv*GK analyzed under native conditions (10 mM ammonium acetate solution).

Coupling native MS to structure- or function-sensitive methods has been helping to characterize the gaseous conformations of proteins. Ion mobility (IM), for example, can be used, not only as a gas-phase separation technique, but also as a method that measures the size and shape of the proteins in the gas-phase [37]. By comparing experimentally obtained collision cross sections (CCS) with theoretically computed values from x-ray or NMR data it is possible to get some insight about the relationship between gas-phase and solution-phase conformations [38,39,40,41]. Nevertheless, the results obtained by IM-MS on well-known proteins, like cytochrome c and ubiquitin, have been conflicting, mainly due to discrepancies in instrumental setups and conditions [42]. As pointed out recently, various instrumental parameters can affect the CCS measurements, thus any correlations between gas- and solution-phase conformations based on IM-MS experiments need to be taken cautiously [43]. Hydrogen/deuterium exchange (HDX) in the gas-phase is another technique that can be used to assess proteins conformational states in the gas-phase [44,45,46]. The level of incorporation of deuterons from the surrounding gas by a protein depends on the surface accessibility of its protons and thus on its conformation. Even though low levels of exchange have been correlated with compact structures and higher levels of exchange with more elongated conformations, the actual mechanisms of exchange in the gas-phase are still not completely understood and more studies are needed to support the validity of such direct correlations [47,48]. Differences in the deuterated gas used, sampling time after ESI of the protein ions and the time allowed for deuterium exchange complicate the interpretation of the available data. Nevertheless, recently developed instrumental setups have delivered results that suggest that solution-phase structural features can be preserved in the gas-phase [48,49,50,51]. Gas-phase fragmentation methods that are specific to surface-exposed regions, such as electron capture dissociation (ECD) and electron transfer dissociation (ETD) have also been extensively used to decipher gas-phase conformations [52,53,54,55,56,57,58,59]. These methods use the fact that capture/transfer of an electron by a multiply charged cation causes dissociation of its backbone with minor excitation of noncovalent bonds. Therefore, observation of separated fragment ions derived from protein backbone cleavage reveal the absence of noncovalent bonding between them and, by default, the putative identification of protein regions with noncovalent interaction [60]. Early ECD studies on cytochrome c have shown that protein regions that interact hydrophobically tend to unfold upon transfer to the gas-phase and their later refolding can lead to stable ion structures that are quite different from the native solution structure [53,54,55]. Further ECD experiments on the three-helix bundle protein KIX indicated preservation of the native solution structure, where electrostatic interactions could compensate for the loss of hydrophobic bonding and stabilize the protein structure in the gas-phase [56]. The conclusions from this and other studies are that the ability of native MS to provide information about the solution-phase structure of a protein depends highly on the extent of intramolecular stabilization by electrostatic interactions [56,57,58]. Recently, ECD fragmentation patterns have been correlated with B-factors from x-ray crystallography, a parameter that represents atomic displacement and is large for protein regions having flexibility and high dynamics [61,62]. While IM and HDX require the acquisition of specialized equipment, top-down features of ECD and ETD experiments are included in many commercially available FT-ICR, orbitraps, and, more recently, TOF instruments. Therefore, ECD/ETD experiments are within reach of those using these instruments to detect and characterize protein-ligand noncovalent interactions, and can be performed to assess the gas-phase conformation of the protein under study. As it is expected that those regions of a protein that fragment readily by ECD/ETD are more flexible and gas exposed, a comparison with the B-factors constitutes a simple way of evaluating if native or near-native conformations have been maintained in the gas-phase. We have, for example, used this tool to study the gas-phase conformation of a potential therapeutic target protein which has been screened against our natural product-based fragment library. This protein is *Plasmodium vivax* guanylate kinase (*Pv*GK), an enzyme that catalyzes the ATP-dependent reversible phosphorylation of GMP/dGMP into GDP/dGDP. By doing so, it provides GDP/dGDP substrate for GTP/dGTP synthesis, which in turn can be used for nucleic acid or cyclic GMP (cGMP) synthesis. Its expression during the liver stage of the *Plasmodium* life cycle suggests it has an important role during this stage and that it may constitute a good drug target [63,64]. The catalytic activity of kinases is generally associated with large conformational changes. Guanylate kinases have a U-shape, which comprises three structural domains: the CORE domain, the LID domain and the GMP-binding domain (GMP-BD). The CORE domain lies in between the LID and the GMP-BD (Figure 2). Previous studies have shown that, during their catalytic cycle, guanylate kinases go from an “open” unbound state through a partially closed intermediate in which GMP is bound to a fully “closed” state in which both GMP and ATP are bound. These substrate-induced conformational changes result from rigid body movements that bring the LID and the GMP-BD domains closer to each other and toward the CORE domain. Interdomain electrostatic interactions stabilize the closed form of the enzyme [65,66]. Depicted in Figure 2a is the crystallographic (PDB 1Z6G) open state of *Plasmodium falciparum* GK (*Pf*GK), which shares 66% sequence identity with *Pv*GK. The partially closed form of *Pv*GK is also shown in Figure 2a (PDB 2QOR), where the ribbons are colored by average residue B-factor. ECD experiments performed with a Bruker SolariX 12T FT-ICR mass spectrometer revealed that *Pv*GK adopts a compact conformation in the gas-phase, similar to the partially closed or closed conformation, even in the absence of substrate binding (Figure 2b). The obtained low percent sequence coverage (7.4%) correlates well with the low B-factors observed throughout the protein structure from PDB 2QOR. However, the fragmentation obtained in the C-terminus of the protein (β8 sheet and α7 helix) indicates that this region is more exposed and accessible to electrons in the gas-phase. Overall, the results show that *Pv*GK assumes a near-native conformation in the gas-phase, where in the absence of water and hydrophobic interactions, electrostatic interactions drive the structure to close down even without substrate binding.

## 3. Detecting Noncovalent Protein-Fragment Interactions

Native MS offers a rapid, sensitive and high throughput method for fragment screening. The amount of protein needed depends on its intrinsic ionization efficiency, the type and concentration of buffer used and the instrumental conditions. Generally, after optimization of experimental and instrumental conditions, protein concentrations in the low micromolar concentration range are used. 2 µM of *Pv*GK, for example, is enough to produce a S/N adequate for screening. If fragments of different molecular weights are pooled in groups of eight and screened by standard ESI, 500 µg of *Pv*GK is enough to screen a library of 1000 fragments. If nano-ESI is used, a 10–20-fold decrease in protein consumption can be achieved. Automation and high-throughput is realized by integrating microfluidic technologies [67]. The commercially available Triversa Nanomate (Advion, Ithaca, NY, USA) platform has, for instance, been used on many screening campaigns [68,69,70,71,72,73,74,75]. This system combines a robot, which aspirates and delivers the samples from multiwell plates with a nano-ESI-microfluidic chip containing 400 nozzles. It offers one-time spray optimization and enhances spray stability and reproducibility with reduced analysis time and no carryover. 

**Figure 3:**
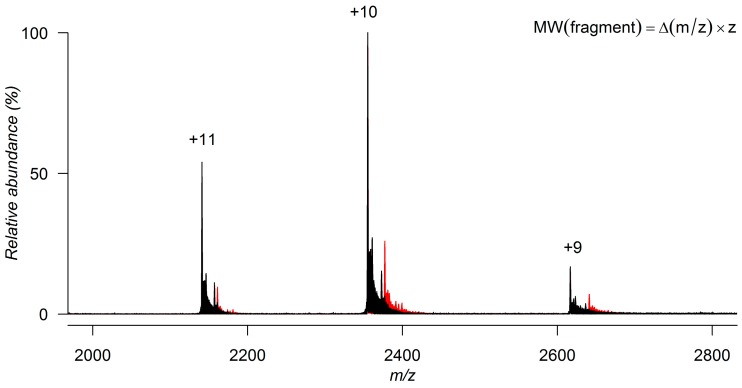
Superimposition of the ESI-MS spectra of *Pv*GK acquired under native conditions (10 mM ammonium acetate solution) before (black) and after incubation with a weakly binding fragment molecule (red). The molecular weight of the binding fragment can be determined by multiplying the difference between the protein-fragment complex peak and free protein peak (Δ*m*/*z*) by the corresponding charge state (*z*).

Native MS provides a direct visualization of all species present in solution. Chemical labelling or crosslinking is unnecessary. The detection of low-affinity ligand binding requires, nevertheless, gentle desolvation and ion transfer conditions to minimize energetic activation and dissociation of the noncovalent protein-ligand complex. To identify fragment hits, the ESI mass spectrum recorded for the protein alone is compared with the spectra acquired after incubation with fragment molecules at 1 to 20 molar equivalents. If a fragment is not binding to the protein in solution, the two spectra are identical and only one species is detected, the protein. If a fragment is binding to the protein, the spectrum shows an additional peak, which corresponds to the protein-ligand complex. For a fragment with a 1:1 binding stoichiometry, this peak is shifted from the peak of the free protein by *m*/*z* units that match the molecular weight of the fragment divided by the corresponding charge state of the protein (Figure 3) [19,73,76].

To confirm the protein-fragment interaction is noncovalent in nature, the cone/skimmer voltage can be slightly increased to observe the disruption of the protein-fragment noncovalent interactions in the gas-phase and the elimination of the complex peak from the mass spectrum. The cone/skimmer voltage determines the kinetic energy of the ions during the transfer from atmospheric pressure to vacuum and therefore affects the energy of the collisions with residual background gas molecules. If the collisional energy transfer from the background gas molecules is high enough, noncovalent bonds are disrupted. This process is known as in-source collision induced dissociation (CID). However, there is a limit to this, since if too much energy is transferred to the gas-phase ions, protein backbone covalent fragmentation occurs. If protein-fragment covalent interactions are still suspected, the sample should be analyzed under denaturing conditions, where all intra and intermolecular noncovalent bonding is disrupted and covalent bonding maintained [73]. 

The observation of statistical multiligand binding indicates the presence of nonspecific noncovalent complexes arising from any gas-phase or in-solution artifactual association. Because specific and non-specific complexes have the same mass, it is not possible to differentiate between them directly from the mass spectrum. Even though different methods have been proposed to separate specific from nonspecific binding based on the distribution of binding stoichiometries [77,78,79], much care must be taken when applying them to fragment binding. These methods tend to work well when the binding affinity for the specific protein-ligand complex is much higher than those observed for nonspecific complexes. However, in the case of fragments, this assumption may not necessarily hold, since the specific and nonspecific complexes may have affinities of the same order in the gas-phase. In fact, the physico-chemical effects described earlier that result from a protein being transferred from solution into the gas-phase also apply to the binding interface between a protein and a ligand. This means that the nature and number of the noncovalent interactions that stabilize a protein-ligand complex in the gas-phase can be different from in the solution. If a ligand is binding to a protein in solution mainly through hydrophobic interactions, the protein-ligand complex most likely dissociates in the gas-phase [71,80,81,82]. In the worst-case scenario, where the complex dissociates completely and cannot be detected, a false negative is generated. On the other hand, if a protein-ligand complex exists in solution due to electrostatic interactions or a combination of electrostatic and hydrophobic interactions, the former stabilize the complex upon transfer into the gas-phase [80,83]. Even though these events disturb the correlation between the relative ion abundances of the different species in the mass spectra with the relative concentrations of the same species in solution, they have an advantage for fragment screening, where the selection of enthalpically-driven fragments delivers higher quality starting points for medicinal chemistry optimization [74,76,80].

## 4. Characterizing Noncovalent Protein-Fragment Interactions

After identifying a set of fragments binding to a particular protein, native MS can also be used as a tool to investigate the selectivity for that protein target, the specificity for a certain protein site and the relative or absolute binding affinities.

When a fragment library is screened against a range of different proteins, the resulting protein-fragment interaction network provides an overview of the number of proteins that each fragment bound to and the number of fragment hits of each protein. Even though fragments that bind to many different proteins may simply be nonspecific or promiscuous binders, they, conversely, could be privileged structures for fragment elaboration into higher selective and potent molecules. Kutchukian et al. have, for example, found that a higher proportion of frequent binders were successfully crystallized with the partner proteins than non-frequent binders [84].

**Figure 4:**
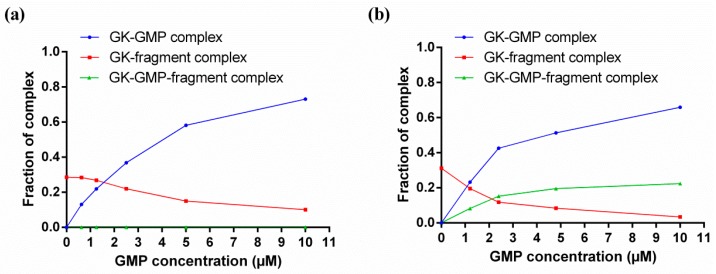
Native MS competitive binding experiments between *Pv*GK natural substrate 5′-guanosine monophosphate (GMP) and a fragment hit, illustrating a situation where (**a**) GMP is competing with the fragment for the same protein-binding site; (**b**) GMP is not competing with the fragment and is binding to a different protein site.

If a known ligand with a known binding site (natural substrate, activator or inhibitor) is available, this can be used to quickly assess the fragment specificity for that site through competitive binding experiments. Native MS competitive binding experiments consist in keeping the protein and fragment concentrations constant, while increasing the known ligand concentrations from 0 until full saturation of the protein, and monitoring the relative abundances of the different species in the mass spectra (Figure 4). If the known ligand is competing with a fragment for the same site, the fraction of the complex with the fragment decreases and the fraction of the complex with the known ligand increases as the known ligand concentration is inc**r**eased, and no ternary complex between the protein, the fragment and the known ligand is observed (Figure 4a). If the known ligand is not binding in the same site as the fragment, as the known ligand concentration increases and the protein becomes saturated, the decrease in the fraction of the complex with the fragment is accompanied by a proportional increase in the fraction of the ternary complex between the protein, the fragment and the known ligand (Figure 4b). A recent example on the application of these kinds of experiments can be found on the work published by Sirtori et al., who used a reference inhibitor known to bind at the ATP binding site of heat shock protein 90 (Hsp90) to narrow down their fragment hit list to ATP-competitive fragments [85]. 

Native MS competitive binding experiments can also be used to qualitatively rank the binding affinity of the different fragment hits [86]. When the equilibrium dissociation constant (K_D_) of the known ligand is known, competition data can be used to deduce the absolute binding affinities of the competing fragments, since the level of displacement of the known ligand from the protein by a competing fragment depends on their respective concentrations and binding affinities for the protein [87,88]. In fact, for low-affinity ligands, competitive binding experiments are a better choice for K_D_ determination than the direct ESI assay or titration methods, as will be discussed next.

Solution-phase equilibria can be maintained during native MS measurements and accurate K_D_ determinations obtained through direct ESI assay or titration methods [7]. The direct ESI assay is performed at a single known initial concentration of protein and ligand. The titration method is normally performed at a fixed concentration of protein and increasing concentrations of ligand. In both methods, the K_D_ is determined from the ratio of total abundance of protein-ligand complex and free (or total) protein ions. While native MS is able to preserve the equilibrium abundance ratio of bound to free (or to total) protein present in solution both during the ESI process and in the gas-phase, it does not mean that it is not prone to physical or chemical processes that alter this ratio. Problems such as nonuniform ionization and detection efficiencies between bound and free protein, protein-ligand complex dissociation, non-specific ligand-protein binding, and ESI-induced changes in solution pH and temperature may arise [14]. These need to be addressed before any correlations between relative abundances of the different species in the mass spectra and the relative concentrations of the same species in solution are made [89]. For fragments, the frequent formation of nonspecific complexes at high concentrations poses a special challenge to accurate K_D_ determinations. To minimize the influence of nonspecific binding, a quick gel-filtration step can be added prior to MS analyses to eliminate excess free fragment present in solution after incubation with the protein [90]. Alternatively, a titration experiment can be performed at different initial protein and fragment concentrations, but identical molar ratios. Herein, the fragment-protein molar ratio should be 1 or otherwise be kept at the lowest level possible [91]. However, if fragment concentrations are not increased in relation to the protein concentration, the occurrence of gas-phase dissociation cannot be detected. This problem is normally diagnosed when the fractional protein occupancy is observed to not tend to 1 when plotted as a function of increasing (and saturating) concentrations of fragment. The experimental fractional protein occupancy at saturation reflects the type of noncovalent interactions involved in protein-fragment binding and can be exploited to select fragments with determined binding characteristics [90].

## 5. Native MS in FBDD: Where Are We?

The usefulness of native MS for FBDD was first demonstrated in 2002 by Swayze et al., who extended the concept of “SAR by NMR” originally published by Fesik and co-workers in 1996 [92] to “SAR by MS” [93]. Native MS was used to screen compound libraries against a subdomain of 23S rRNA. The hits were ranked by binding affinity and two classes of motifs with interesting structure-activity relationship (SAR) trends were identified. Native MS competitive binding experiments between the different ligand classes were performed and the results obtained used to rationally synthesize fused compounds. One compound displayed a 20-fold increase in binding affinity as well as good functional activity. Noteworthy, the authors had failed to find hits when screening was carried out using conventional HTS methods [93]. Later, Ockey et al. applied the same “SAR by MS” approach to find novel inhibitors of stromelysin [94]. First, by deconstructing known inhibitors into fragments, studying their binding to the target protein separately or in combination to assess common or distinct binding sites and binding affinities, the suitability of the approach was established. Second, by screening a small library of novel fragments and obtaining SAR, a novel lead fragment was identified. Third and last, by linking a fragment, whose binding site was identified using native MS competitive binding experiments, with a fragment from a known inhibitor, a novel inhibitor of stromelysin was developed [94]. Native MS has also been combined with dynamic combinatorial chemistry [95]. In order to efficiently identify the best combination of building blocks in a dynamic combinatorial library, Poulsen et al. used native MS to directly identify the binding ligands formed by hydrazone exchange in the presence of the target protein (bovine carbonic anhydrase II, or bCA II) [95]. The utility of native state MS in FBDD has again been demonstrated in the work conducted by Moore et al., who used it as tool to validate fragment binding to BIR2-BIR3 motifs (bacculoviral inhibitory repeats) of X-linked inhibitor of apoptosis protein (XIAP) and assign selective binding to either BIR2, BIR3 or both. Native MS competitive binding experiments enabled the ligand-binding site within the BIR3 motif to be localized [96]. To determine the capacity of native MS to identify the same fragment hits as X-ray crystallography, Drinkwater et al. tested a set of 12 fragments, which included four positive and eight negative hits, as determined by x-ray crystallography, against human phenylethanolamine *N*-methyltransferase (PNMT). The pilot study identified the same fragment hits as X-ray crystallography along with two additional fragment hits and concluded that native MS could be used as a fast and efficient method for fragment screening prior to characterization of binding mode by X-ray crystallography [72]. In a FBDD campaign carried by Hannah et al., native state MS was used to automatically screen a library of 350 fragments against Hsp90 using the Triversa Nanomate (Advion, Ithaca, NY, USA) platform. This screening needed just 2 mg of desalted protein to test the whole fragment library in duplicate and afforded a 12.5% hit rate. On the other hand, screening of a library of 60 fragments previously selected by virtual screening generated a 50% hit rate [73]. Another fully automated high-throughput screening method was developed and validated by Maple et al. [74]. Using the Triversa Nanomate platform, detection and direct quantification of fragment binding from µM to mM K_D_ range was accomplished after experimental and instrumental optimization. The method was applied to screen a targeted library of 157 compounds against the anti-apoptotic protein bcl-x_L_. The screen took 6 h and consumed less than 1 mg of protein [74]. Recently, Vu et al. used native MS to screen a natural product-based fragment library of 331 members against *Plasmodium falciparum* 2′-deoxyuridine-5′-triphosphate nucleotidohydrolase (*Pf*dUTPase). Securinine and its hydroxylated analog were identified as interesting hits. To obtain SAR, 5 other analogs were tested. The molecules were found to enhance *Pf*dUTPase activity and inhibit *Plasmodium falciparum* asexual erythrocytic stage and stage V gametocytes [97]. Woods et al. have also, recently, published the results of a FBDD campaign designed to find new inhibitors of human carbonic anhydrase II (hCA II) [75]. In a first stage, primary screening of a 720-member fragment library was performed by surface plasmon resonance (SPR). The seven identified hits were then confirmed by native MS and x-ray crystallography. A good correlation between the different biophysical methods employed was found. In a second stage, a library of 70 compounds was screened in parallel by native MS and SPR. Overall, an 83% agreement between the two screening methods was found. At this stage, x-ray crystallography was only performed for selected fragment hits [75]. 

Even though not many examples that describe the application of native MS in FBDD can be found in the literature (Table 1), native MS is now a well-established technique with proven utility for FBDD. Its high sensitivity, simplicity, speed, wide dynamic range, low protein and ligand consumption, and possibility of automation and high-throughput place it in a competitive position with other commonly used biophysical methods for primary screening, such as STD-NMR and SPR. For example, if Maple et al. had performed their primary screening by STD-NMR, approximately 20 times more protein and 250 times more ligand would have been consumed, and the screening taken over 15 times longer [74]. However, the application of native MS in FBDD is limited by the size of the protein that can be screened, which is determined by the actual achieved mass resolution. This depends on the type of mass analyzer, natural isotopic distribution and level of desolvation and adduct formation with buffer and salts [98]. For proteins larger than 80 kDa, resolving fragment binding by native MS may become very challenging. Native MS in FBDD has also been limited to soluble proteins. Nevertheless, the exciting recent progress in the field of membrane proteins will certainly broaden the repertoire of protein targets screened by native MS in the future [99,100]. The development of innovative ionization techniques such as laser-induced liquid bead ion desorption (LILBID) and of solubilization systems that can be released following collisional or infrared laser activation, including micelles, bicelles, liposomes, amphipols and nanodiscs, have all contributed to the demonstration that the analysis of intact membrane protein-ligand complexes in the gas-phase is feasible [100,101,102,103]. Native MS is, like any other biophysical method used to probe protein-fragment interactions, also prone to false positives and false negatives. Therefore, it is not usually used as a stand-alone method but integrated with orthogonal techniques. Indeed, each screening method operates through different biophysical principles and the analyses are often performed at different conditions using different hit definition criteria. Sciebel et al. have, for instance, found a low overlap of putative fragment hits, which were identified by screening a 361-member fragment library against endothiapepsin using six different screening methods (biochemical assay, reporter-displacement assay, STD-NMR, native MS, thermophoresis and thermal shift assay) [104]. Later, a comprehensive crystallographic screen of the same library against the same protein target found that 44% of the identified hits had never been detected by any of the previously used biophysical screening methods [105]. While the different screening techniques may bias the hits identified towards determined physico-chemical properties depending on the protein target [84,106], this study definitely demonstrates the value of using the existing biophysical methods in parallel rather than in tandem. Native MS provides direct visualization of fragment binding and it is also advantageous for post-screening characterization of putative fragment hits. Binding stoichiometry and SAR can be obtained and competitive binding experiments can quickly assess fragment specificity for a particular binding site. Although K_D_ determinations by direct-ESI assay or titration still often need to be validated by an orthogonal method such as ITC, competitive binding experiments generally provide accurate values. Given the significant improvements in mass spectrometry hardware and software in recent years, it is expected that native MS will become more available to researchers and be seen more often integrated into FBDD campaigns, alongside other well-established biophysical methods.

## Figures and Tables

**Table 1:** Applications of native MS found within FBDD campaigns from the literature.

Protein Target	Library Size	Primary Screening	Hit Validation	Relative Binding Affinty	Absolute Binding Affinity	Binding Specificity	Reference
Subdomain of 23S rRNA	NS	×	−	×	−	×	[93]
Stromelysin	15	×	−	−	×	×	[94]
bCA II	10	×	−	−	−	−	[95]
XIAP	NS	−	×	−	−	×	[96]
PNMT	12	−	×	−	−	−	[72]
Hsp90	350	×	−	−	−	−	[73]
Hsp90	60	−	×	−	−	−	[73]
Bcl-x_L_	157	×	−	−	×	−	[74]
*Pf*dUTPase	331	×	−	×	−	−	[97]
hCA II	720	−	×	×	−	−	[75]
hCA II	70	×	−	−	−	−	[75]
Endothiapepsin	361	×	−	−	−	−	[104,105]

NS: Not specified.
